# Spiritual Experience in Dementia From the Health Care Provider Perspective: Implications for Intervention

**DOI:** 10.1093/geroni/igab046.1791

**Published:** 2021-12-17

**Authors:** Jennifer Palmer, Michelle Hilgeman, Tracy Balboni, Sara Paasche-Orlow, Jennifer Sullivan

**Affiliations:** 1 Center for Healthcare Organization and Implementation Research, VA Boston Healthcare System, Boston, Massachusetts, United States; 2 Tuscaloosa VA Medical Center, Tuscaloosa, Alabama, United States; 3 Harvard Medical School, Boston, Massachusetts, United States; 4 Hebrew SeniorLife, Roslindale, Massachusetts, United States; 5 VA Providence Medical Center LTSS COIN and Brown University, Providence, Rhode Island, United States

## Abstract

Spiritual care seeks to counter negative outcomes from spiritual distress and is notably needed in dementia. Such care needs disease-appropriate customization. Employing “cognitive apprenticeship” theory’s focus on learning from contrast, we explored spiritual needs salient within dementia as related to other disease states; we aimed to inform future dementia-focused spiritual care design. Accordingly, we conducted semi-structured qualitative interviews with 24 providers who serve older adults inclusive of persons with dementia. We sampled participants purposively by discipline (chaplains, nursing staff, social workers, activities professionals) and religious tradition (for chaplains). Our interview guide inquired about the nature of spiritual needs in dementia and stakeholders’ roles in addressing them. Hybrid inductive/deductive thematic analysis was employed. A thematic structure emerged with two themes: 1) spiritual experience in dementia compared to other medical conditions (sub-themes: the salience of (a) fear; (b) loss of self; (c) dementia’s progressive and incurable nature; (d) dementia’s impact on accessing faith); and 2) the need for spiritual intervention at the mild stage of dementia (sub-themes: (a) awareness in mild dementia and its influence on spiritual distress; (b) a window of opportunity). These findings pointed to possibilities for the “what” of spiritual needs and the “who” and “when” of implementing spiritual care. Implications included the imperative for dementia-specific spiritual assessment tools, interventions targeting fear and loss early in the disease, and stakeholder training. Researchers should study the “how” of dementia-appropriate spiritual care given recipients’ cognitive and linguistic challenges. Conjointly, these efforts could promote the spiritual well-being of persons with dementia worldwide.

